# Commentary: how will interventional oncology navigate the “valleys of death” for new medical devices?

**DOI:** 10.1259/bjr.20170643

**Published:** 2018-01-19

**Authors:** Ricky A Sharma, Lucio Fumi, Riccardo A Audisio, Alban Denys, Bradford J Wood, Francesco Pignatti

**Affiliations:** 1 Department of Oncology, NIHR Oxford Biomedical Research Centre, University of Oxford, Oxford, UK; 2 NIHR University College London Hospitals Biomedical Research Centre, UCL Cancer Institute, London, UK; 3 Wyfold Medical Consultancy, Wyfold, UK; 4 University of Liverpool, St Helens Teaching Hospital, St. Helens, UK; 5 Department of Radiology and Interventional Radiology, CHUV University Hospital, Lausanne, Switzerland; 6 Center for Interventional Oncology, National Cancer Institute and NIH Clinical Center, National Institutes of Health, Bethesda, MD, USA; 7 European Medicines Agency, London, UK

## Abstract

Whereas clinical trials of cancer drugs have methodological standards and conventional primary endpoints, these are not necessarily applicable to the clinical development of loco-regional treatments and new medical devices. The current challenge is to generate high-level clinical evidence for loco-regional treatments to define the benefits for patients. In this article, we argue that, to generate convincing evidence of clinical efficacy and safety, the collective coherence of the entire data package is often more important than the primary endpoint of one clinical trial. We also propose that, when a comprehensive clinical data package is not feasible, limited clinical data can be supplemented with other types of evidence. Emerging life science companies often define the “valley of death” after securing initial investment to translate an early medical device concept to a development stage that is attractive to funders. Unfortunately for this industry, there is a second “valley of death” if the focus and goal is only regulatory approval, to the neglect of clinical acceptance and reimbursement. For the emerging specialism of interventional oncology, it is critical to plan a clear line of sight for each new medical device to avoid the valleys of death and to demonstrate the clinical benefit. Increased international guidance to establish realistic yet convincing standards in this area may avoid attrition of potentially beneficial devices and therapeutic procedures in the valleys of death.

## The current issues

Clinical trials of cancer drugs have methodological standards and conventional primary endpoints, particularly overall survival and progression-free survival. These standards are based on the industry’s experience of a line of sight for a new drug, with an understanding of the regulatory challenges that will be encountered to achieve the licensing goal. Clinicians play a key role in defining new regulatory standards and justifying clinically relevant endpoints.^1–4^

The standards and endpoints for new drugs are not necessarily applicable to the clinical development of loco-regional treatments and new medical devices. Clinicians in the disciplines of surgical oncology, radiation oncology and interventional oncology (IO) recognize that the current challenge for these specialties is to generate high-level clinical evidence for loco-regional treatments to define the benefits for patients.^2, 3,5^ There is a need for standards that are proportionate, reasonable and pragmatic, and for clinical studies with realistic and patient-centred endpoints. Consistent with a recent article on the licensing of new drugs,^1^ we argue that, to generate convincing evidence of clinical efficacy and safety to regulators, the collective coherence of the entire data package is often more important than the primary endpoint of one clinical trial. Furthermore, when a comprehensive clinical data package is not feasible, limited clinical data can be supplemented with other types of evidence (*e.g.* from clinical registries or from observational studies).

An example in point is selective internal radiotherapy using yttrium-90 microspheres. One manufacturer of this class of medical device invested in long-term, phase III, randomized controlled trials with conventional medical oncology endpoints (overall survival and progression-free survival) as primary endpoints for studies in patients with metastatic colorectal cancer or hepatocellular carcinoma.^6, 7 ^Although these studies did not reach their primary endpoints, they demonstrated the importance of considering the entire package of clinical data when discussing the safety and efficacy of a medical device with patients (*e.g.* control of liver disease for a liver-directed therapy when added to standard therapy, and quality of life following a liver-directed therapy as an alternative to systemic therapy). An example of a medical device with FDA premarket approval which has not been adopted widely is the AspireAssist weight-loss device^8 ^This device, which uses a surgically-placed tube to drain a portion of the stomach contents after every meal, is an example of an approved device for which health professionals may be waiting for more evidence to be published before advocating the technology more widely.

A recent trend in IO has been to divert resources away from clinical trials of medical devices towards the collection of more heterogeneous “real world” data in clinical registries.^2, 9^ This can be justified, in some cases, by the realization that formal clinical trial pathways can take a decade or more to address endpoints such as overall survival. During this long timescale, the technology being studied or the standard of care for that disease may have evolved, rendering the study obsolete. Assuming the quality of the data being collected in a “real world” setting is acceptable, we wish to emphasize that registry data can generally augment but not substitute for high-quality clinical trial data to establish safety and efficacy. Registries may address specific uncertainties in a timely and pragmatic way when randomized controlled clinical trials are not feasible.

**Figure 1.**
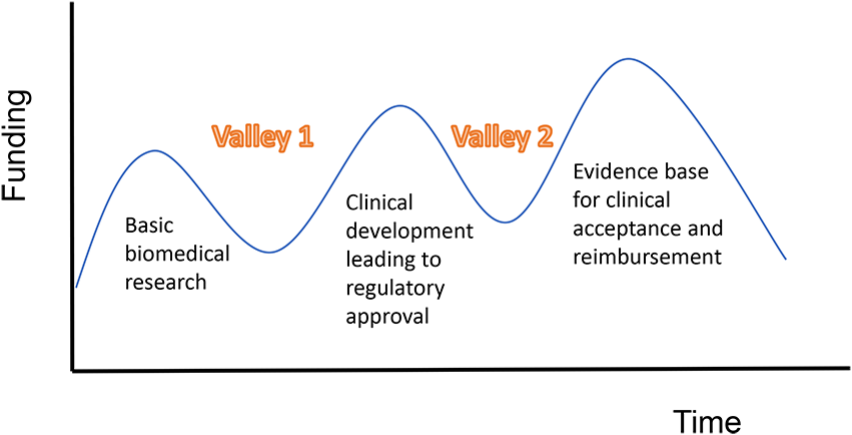
The valleys of death for new medical devices. Life science companies often define the “valley of death” as securing the initial investment to translate an early medical device concept to a clinical development stage that is attractive to funders. There is also a second “valley of death” potentially following regulatory approval, if sufficient clinical evidence is not demonstrated for clinical acceptance, cost-effectiveness and reimbursement.

Diverting funds away from clinical trial development is, unfortunately, only the tip of the iceberg in the current threat to the medical device industry. Emerging life science companies often define the “valley of death” after securing initial investment to translate an early medical device concept to a development stage that is attractive to funders.^10^ Unfortunately for this industry, there is a second “valley of death” if the focus and goal is only regulatory approval, to the neglect of clinical acceptance and reimbursement (Figure 1). In Europe, medical devices require a conformité européenne (CE) mark. Having obtained a CE mark, companies are often unable to find users for the product due to lack of clinical evidence supporting its clinical benefit or cost effectiveness. In the USA, the US Food and Drug Administration (FDA)’s 510(k) submission for “equivalence” to a predicate device (one that has been cleared by the FDA or marketed before 1976) does not mandate clinical data. Devices with higher risk require a premarket application, which does require clinical data, but generally less than submissions for new cancer drugs.

The current situation is similar to the fundamental problem described by Sung et al^11^ over a decade ago when they described the blocks to translation of basic science discoveries in to high quality clinical studies due to high costs, lack of funding, regulatory burdens, fragmented infrastructure, incompatible databases and a shortage of qualified investigators and willing participants. Although specific to the USA, some of the recommendations made by Sung et al are broadly relevant to the issues described in this article for interventional oncology; some of the suggestions are incorporated in to our recommendations (see below).

Payers and other public or private organizations may also take an active role to generate evidence to inform treatment and reimbursement decisions. The US Centers for Medicare and Medicaid Services have recently implemented “Merit-based Payment Systems” and “Alternate Payment Models”, which may empower outcomes analyses for new and old medical technologies.

## Recommendations

In order to address some of these issues, we recommend that there should be a recognition that the outcomes of IO clinical trials depend on factors such as the manual abilities of the operator, the number of cases done per year by a centre, differences in practice in different centres, dependency on sophisticated medical devices with complex learning, diversity of medical devices used in different centres and rapid technological evolution of techniques and devices. Providers of interventional oncology should be encouraged to ensure education of their staff to apply clinical evidence to their own clinical practice and decision making. Standards to generate high-level clinical evidence should be similar in aims to those accepted for new drugs in oncology. However, they should acknowledge the different regulations, challenges, specificities and flexibility needed for IO trials. Regulations should be standardized and streamlined, allowing information to be accessed by both investigators and the general public. Standards must be proportionate, reasonable, pragmatic, and they should help the clinical studies to achieve realistic and clinically important objectives. Financial conflicts of interests among investigators, institutions and healthcare providers should be transparent.

Overall survival is still the gold standard as a primary endpoint for drug studies, but in practice it is often not the ideal choice for IO studies. Reasons for this include the long survival of patients, evolution of the technology during that timescale, and diverse, sequential treatments making it very difficult to associate overall survival with one specific treatment.^6^ The choice of primary endpoint needs therefore to consider reliable and readily measurable intermediate efficacy endpoints. Collections of endpoints such as overall response rate, tumour response, depth of response, time to progression, organ-specific progression-free survival, might be good candidates alongside quality of life, activities of daily living and other patient-reported outcomes, and health economics assessments.

Finally, to the users of the devices, *i.e.* the physicians and the multidisciplinary teams, we advise them to explicitly ask the manufacturers for evidence of efficacy before they consider changing their clinical practice. Providers of healthcare and professional societies should promote and support a research culture that mandates a certain level of clinical evidence for all the health services being provided.

## Conclusions

For the emerging specialism of interventional oncology, it is critical to plan a clear line of sight for each new medical device to avoid the “valleys of death” and to demonstrate the clinical benefit. Increased international guidance to establish realistic yet convincing standards in this area may avoid attrition of potentially beneficial devices and therapeutic procedures in the “valleys of death”.
